# Supplementary material for: Offshore transport of particulate organic carbon in the California Current System by mesoscale eddies
Source: Nat Commun. 2019 Oct 30;10:4940. doi: 10.1038/s41467-019-12783-5 (PMC6821764; doi:10.1038/s41467-019-12783-5)
Supplement: Supplementary file 2 — Supplementary Information [file 41467_2019_12783_MOESM2_ESM.pdf]

SUPPLEMENTARY INFORMATION

Offshore transport of particulate organic carbon in the California Current System by mesoscale eddies

Caitlin M. Amos[1], Renato M. Castelao[1,*] and Patricia M. Medeiros[1]

[1]Department of Marine Sciences, University of Georgia, USA

Marine Sciences Building, 325 Sanford Drive, Athens, GA 30602

*Corresponding author: castelao@uga.edu

Supplementary Figures

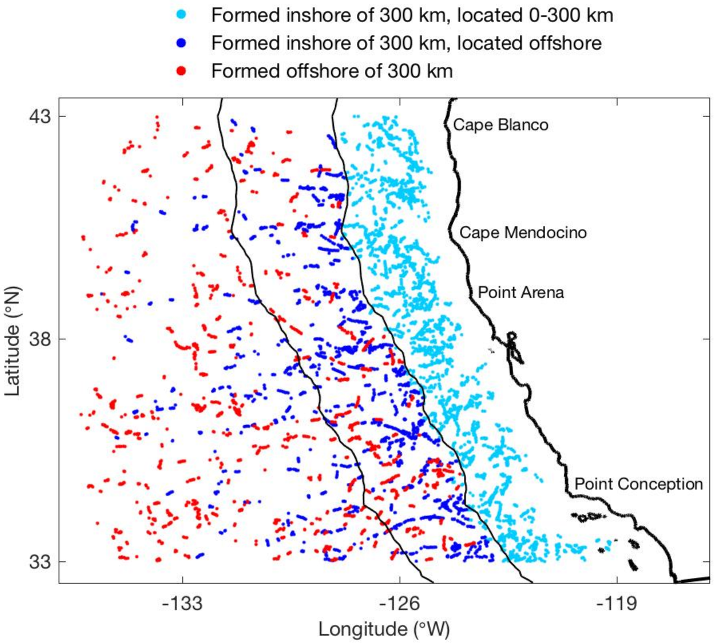

Supplementary Figure 1. Locations of cyclonic eddy occurrences in the California Current System under clear-sky conditions. Cyclonic eddy occurrences identified in the eddy dataset[1] with at least 90% particulate organic carbon pixel coverage inside one eddy radius and 75% coverage inside two radii. Thin black lines mark 300 and 600 km from the coast.

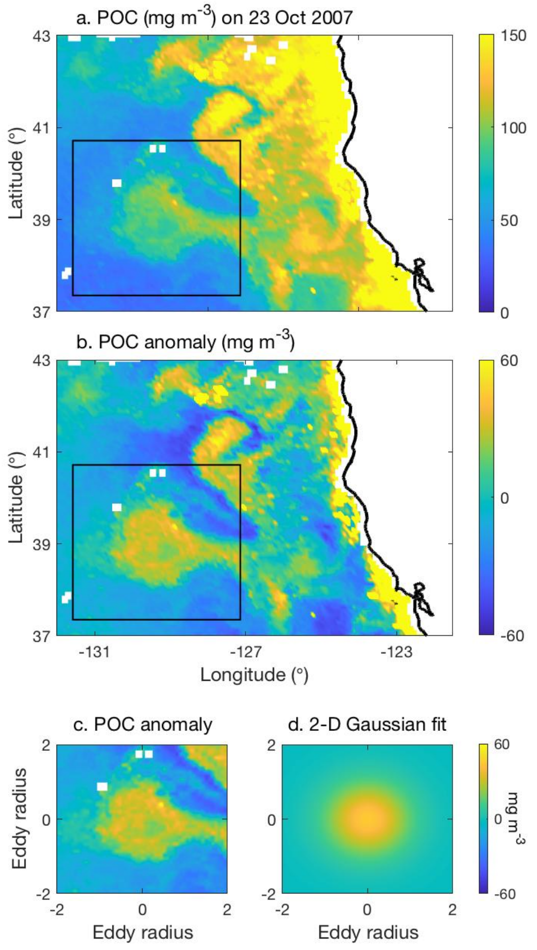

Supplementary Figure 2. Procedure for extracting the particulate organic carbon anomaly inside eddies. (**a**) Particulate organic carbon (POC) and (**b**) POC anomaly in the California Current System on 23 October 2007. Black box marks the region within 2 by 2 eddy radii from the eddy center, as identified by altimetry[1]. (c) POC anomaly extracted within the black box in panel b. (**d**) POC anomaly based on 2-D Gaussian fit[2] to the field in panel c (see Methods for details).

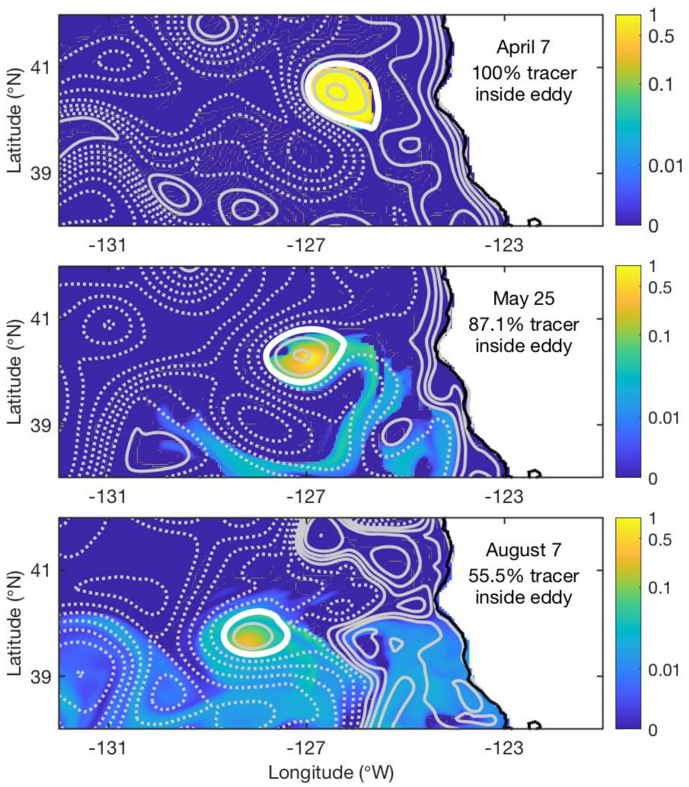

Supplementary Figure 3. Model simulation of eddy transporting coastal water. Example of model simulation of cyclonic eddy using a passive tracer to track eddy lateral transport. Sea surface height is shown by grey contours at 4 cm intervals. Solid contours are negative. Thick white circle marks the boundary of the eddy in each snapshot. The fraction of the tracer that is located in the eddy interior (top 400 m) as the eddy propagates westward is also given.

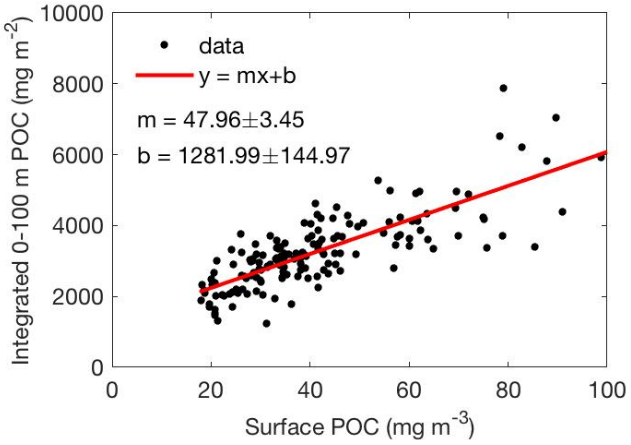

Supplementary Figure 4. Relationship between in situ surface particulate organic carbon and integrated particulate organic carbon in top 100 m. In situ particulate organic carbon concentrations measured in the California Current System between 2006-2016[3] integrated from the surface to 100 m depth (mg m^{-2}) and correlated with concentrations at the surface (mg m^{-3}). Red line shows the linear fit (see Methods).

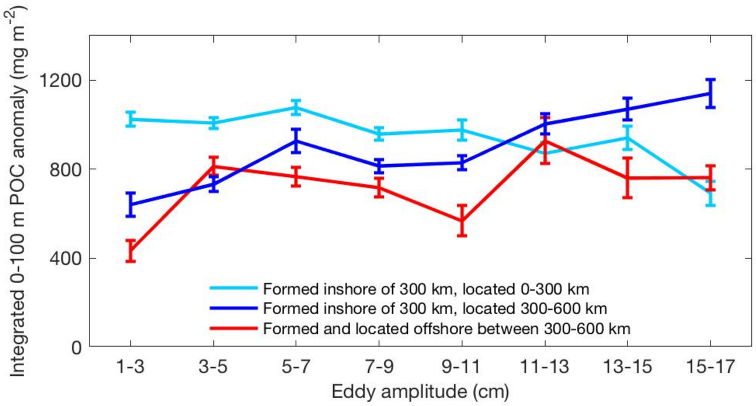

Supplementary Figure 5. Integrated particulate organic carbon anomaly in top 100 m. Mean integrated particulate organic carbon anomaly (mg m^{-2}) in top 100 m and standard error within one eddy radius calculated using the linear relationship shown in Supplementary Fig. 4 and grouped by eddy amplitude.

Supplementary Table 1. General characteristics of cyclonic eddies with lifetime longer than 4 weeks generated within 600 km from the coast in the California Current System (1993-2015) from an existing global eddy dataset[1].

	Cyclones generated and located 0-300 km from the coast	Cyclones generated 0-300 km and located 300-600 km from the coast	Cyclones generated and located 300-600 km from the coast
Number of eddies	110	138	121
Amplitude (cm)	5.95 ± 2.94	8.42 ± 4.06	6.77 ± 3.70
Radius (km)	63.77 ± 19.29	83.02 ± 24.88	72.86 ± 24.25
Rotational speed (cm s^{-1})	17.51 ± 5.34	19.42 ± 6.23	16.71 ± 5.88

Supplementary References

1 Chelton, D. B., Schlax, M. G. & Samelson, R. M. Global observations of nonlinear mesoscale eddies. *Prog. Oceanogr.* **91**, 167-216 (2011).

2 Yuan, Y. & Castelao, R. M. Eddy-induced sea surface temperature gradients in Eastern Boundary Current Systems. *J. Geophys. Res. Oceans* **122**, 4791-4801 (2017).

3 Aluwihare, L. Particulate organic carbon and nitrogen measurements at selected depths in the water column in the CCS region since 2006 - 2016 (ongoing). Environmental Data Initiative https://doi.org/10.6073/pasta/20c05dd205be2225ecb32a5fede1c36c (2018).
